# Optimisation of Protein Extraction from Medicinal Cannabis Mature Buds for Bottom-Up Proteomics

**DOI:** 10.3390/molecules24040659

**Published:** 2019-02-13

**Authors:** Delphine Vincent, Simone Rochfort, German Spangenberg

**Affiliations:** Agriculture Victoria Research, AgriBio, Centre for AgriBioscience, Bundoora, Victoria 3083, Australia; simone.rochfort@ecodev.vic.gov.au (S.R.); german.spangenberg@ecodev.vic.gov.au (G.S.)

**Keywords:** urea, guanidine-HCl, trypsin digestion, nLC-MS/MS, apical bud, trichome

## Abstract

Medicinal cannabis is used to relieve the symptoms of certain medical conditions, such as epilepsy. Cannabis is a controlled substance and until recently was illegal in many jurisdictions. Consequently, the study of this plant has been restricted. Proteomics studies on *Cannabis sativa* reported so far have been primarily based on plant organs and tissues other than buds, such as roots, hypocotyl, leaves, hempseeds and flour. As far as we know, no optimisation of protein extraction from cannabis reproductive tissues has been attempted. Therefore, we set out to assess different protein extraction methods followed by mass spectrometry-based proteomics to recover, separate and identify the proteins of the reproductive organs of medicinal cannabis, apical buds and isolated trichomes. Database search following shotgun proteomics was limited to protein sequences from *C. sativa* and closely related species available from UniprotKB. Our results demonstrate that a buffer containing the chaotrope reagent guanidine hydrochloride recovers many more proteins than a urea-based buffer. In combination with a precipitation with trichloroacetic acid, such buffer proved optimum to identify proteins using a trypsin digestion followed by nano-liquid chromatography tandem mass spectrometry (nLC-MS/MS) analyses. This is validated by focusing on enzymes involved in the phytocannabinoid pathway.

## 1. Introduction

*Cannabis sativa* plants have been cultivated and used as a food crop as well for textiles, ropes, paper and medicinal applications for millennia in Asia [1]. Its many uses have since spread worldwide, and it was incorporated to Western medicine in 1938, followed by a rapid adoption among the medicinal community [2]. By the end of the nineteen century, cannabis therapeutical applications had diminished due to the development of more efficient alternative therapies and analgesics, in particular opiates [2]. The beginning of the 20th century saw the surge of cannabis use as a recreational substance in Western countries, which led to the Cannabis Tax Act in 1937 and abolished its use in medicine in the U.S.A. [1]. In 1961, an international treaty called the Single Convention on Narcotic Drugs (http://www.unodc.org) was enacted to prohibit production and supply of specific drugs, including cannabis, with provision made to grant licences for medical treatment and research. Within the framework of this treaty, since 1967 the Australian Narcotic Drugs Act (www.odc.gov.au) has regulated the cultivation of cannabis for medicinal and related scientific purposes. In 1989, the Therapeutic Goods Act was established to ensure that therapeutic goods abide to Australian standards and are promptly accessible (www.tga.gov.au). The Narcotic Drugs Act was amended in 2016 (www.legislation.gov.au) and again very recently in 2018 with the Narcotic Drugs Amendment (Cannabis) Regulations (www.legislation.gov.au) to permit exports of medicinal cannabis products. In 2016, Victoria was the first Australian jurisdiction to legalise access to medicinal cannabis by enacting the Medicinal Cannabis Act 2016 (www2.health.vic.gov.au).

Phytocannabinoids are the best-known active components of *C. sativa*. The first cannabinoid, cannabidiolic acid (CBDA), was isolated in 1940 [3]. The most psychologically active cannabinoid delta9-tetrahydrocannabinolicacid (THCA) was identified in 1967 [4]. Since then, close to 500 compounds have been described [5], including 70 cannabinoids among which are cannabinolic acid (CBNA), cannabigerolic acid (CBGA) and cannabinodiolic acid (CBNDA). The biosynthetic pathway of phytocannabinoids from *C. sativa* was elucidated in 2007 [6]. The main enzymes are 3,5,7-trioxododecanoyl-CoA synthase (OLS, a polyketide synthase) and olivetolicacid cyclase (OAC) acting in succession to convert hexanoyl-CoA into olicetolic acid (OLA), geranylpyrophosphate:olivetolate geranyltransferase (GOT) which catalyses the alkylation of OLA with geranyldiphosphate leading to the formation of CBGA, THCA synthase (THCAS) converts CBGA to THCA, while CBDA synthase (CBDAS) forms CBDA and CBCA synthase (CBCAS) produces CBCA [7].

Due to legislative difficulties, scientific studies on cannabis are lagging behind compared to other economically valuable crops, particularly in the field of proteomics. In the few proteomics reports found, most dealt with nonreproductive organs from immature cannabis plants, such as roots [8] and hypocotyls [9], or processed seeds from hemp [10,11]. As far as we know, only Raharjo and colleages in 2004 published proteomics results on flowers and glands from cannabis plants [12], and ten years later, Happyana produced his PhD thesis on cannabis trichomes [13]. Sample preparation for protein extraction varied in these studies. Some reported a precipitation step in a trichloroacetic acid (TCA)/acetone solution followed by resuspension of the washed pellet in a urea-based buffer [9,12]. Other reports applied an initial powder homogenisation in a Tris-based buffer [10] or a sucrose solution [11] followed by TCA/water precipitation and resuspension in a Tris buffer [10] or a urea buffer [11]. Finally, a phase partition method including homogenisation in Tris-buffer phenol, followed by precipitation in ammonium acetate/methanol with resuspension of the rinsed pellet in a urea-based buffer has been used [8,13]. Protein extracts were then analysed by two-dimensional electrophoresis (2-DE) [8,9,11,12], one-dimensional polyacrylamide gel electrophoresis (1-D PAGE) [9,10], or two-dimensional (2-D) liquid chromatography (LC) followed by tandem mass spectrometry (MS/MS) [13]. None of these studies have used a buffer made of guanidine hydrochloride (also called guanidium chloride), a strong chaotrope reagent more stable than urea which may cause carbamylation of proteins when slightly heated [14]. Guanidine-HCl was proven effective for shotgun proteomics of whole yeast lysate [15], glycoproteins [16], as well as top-down proteomics of milk proteins [17,18,19].

As far we know no attempt was made at optimising the recovery of proteins from cannabis reproductive tissues. To this end, we designed a study where six protein extraction methods were devised and tested on apical bud tissues from medicinal cannabis plants using three biological replicates and two technical replicates. Two of these extraction methods were also tested on isolated trichomes. Protein extracts were further trypsin-digested into peptides for protein identification purpose. Our assessment was based on liquid chromatography–mass spectroscopy (LC-MS) profiles of intact proteins, nLC-MS/MS profiles of tryptic peptides, protein identification results and statistical analyses. We exemplify our observations on enzymes involved in the phytocannabinoid biosynthesis pathway. Optimisation of protein extraction is critical to achieve good coverage of the proteome and key in integrating proteomics with the other omics strategies including genomics and metabolomics. Together these techniques will better define gene function and allow for genomic selection or genome editing to produce cannabis strains with specific traits tailored for particular medical applications.

## 2. Results and Discussion

**Figure 1:**
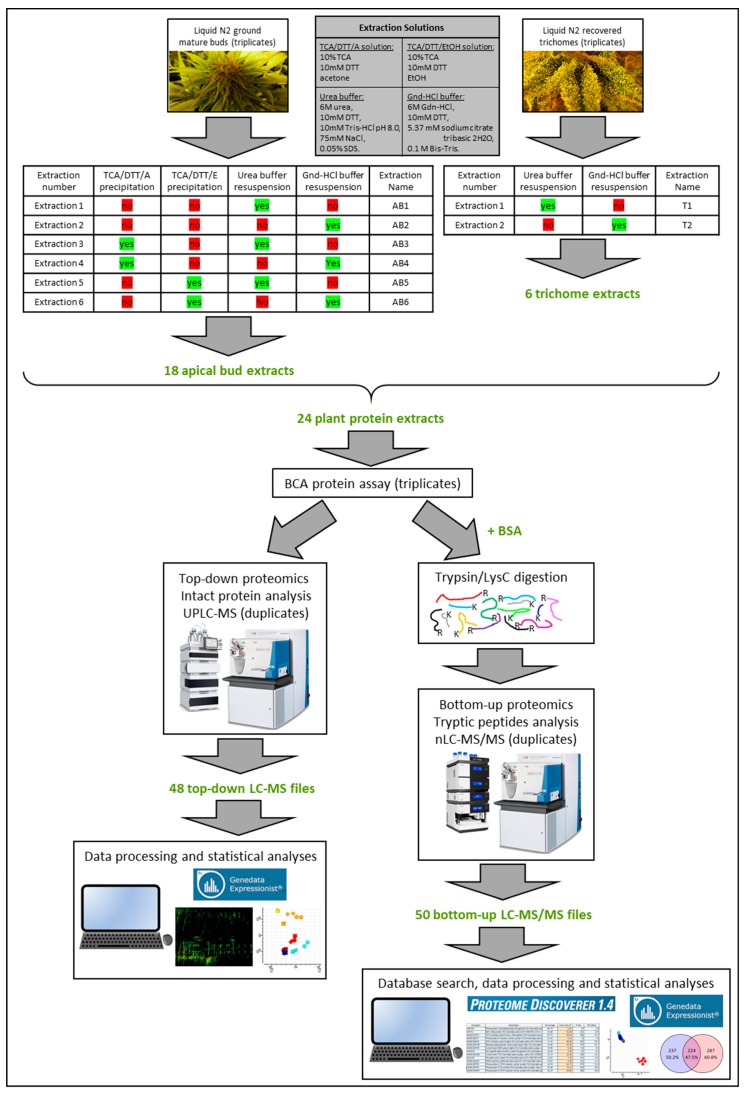
Experimental design.

This experiment aims at optimising protein extraction from mature reproductive tissues of medicinal cannabis. A total of six protein extractions were tested with methods varying in their precipitation steps with the use of either acetone or ethanol as solvents, as well as changing in their final pellet resuspension step with the use of urea- or guanidine-HCl-based buffers (Figure 1). 

The six methods were applied to liquid N_2_-ground apical buds. Trichomes were also isolated from apical buds. Because of the small amount of trichome recovered, only the single step extraction methods 1 and 2 were attempted. Extractions were performed in triplicates. Extraction efficiency was assessed both by intact protein proteomics and bottom-up proteomics each performed in duplicates. Rigorous method comparisons were then drawn by applying statistical analyses on protein and peptide abundances, linked with protein identification results.

### 2.1. Intact Protein Analysis

The intact proteins of the 18 apical bud extracts and the six trichome extracts were separated by ultraperformance liquid chromatography (UPLC) and analysed by electrospray ionisation-mass spectrometry (ESI-MS) in duplicates. LC-MS profiles are complex with many peaks along both retention time (RT) in min and *m*/*z* axes, particularly between 5 and 35 min and 500 and 1300 *m*/*z* (Supplementary Figure S1). Prominent proteins eluted late (25–35 min), probably due to high hydrophobicity, and within low *m*/*z* ranges (600–900 *m*/*z*), therefore bearing more positive charges. Outside this area, many proteins eluting between 5 and 25 min were resolved in samples processed using extraction methods 2, 4 and 6, irrespective of tissue types (apical buds or trichomes). Protein extracts from apical buds and trichomes overall generated 26,892 intact protein LC-MS peaks (ions), which were then clustered into 5,408 isotopic clusters, which were in turn grouped into 571 proteins of up to 11 charge states. The volumes of all the peaks comprised into a group were summed and the sum was used as a proxy for the amounts of the intact proteins. Statistical analyses were performed on the summed volumes of the 571 protein groups.

**Figure 2:**
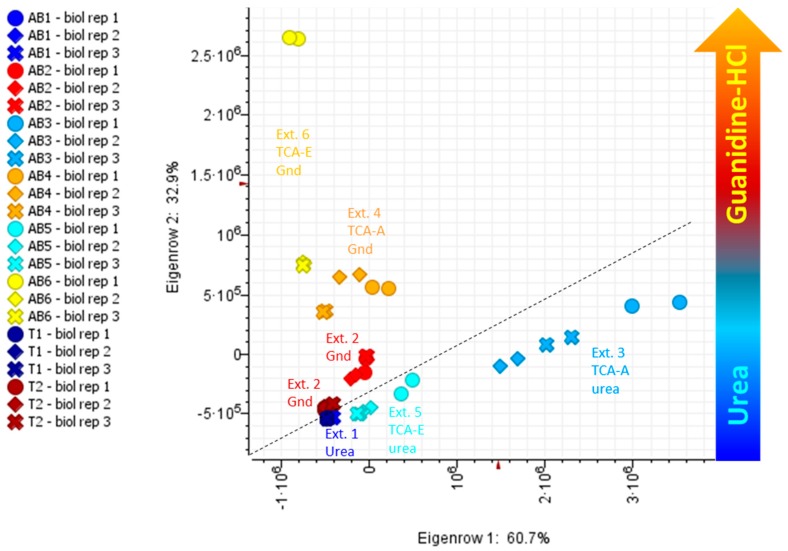
Principal component analysis (PCA) using intact protein data. AB, apical buds; T, trichomes; 1–6, extraction methods 1 to 6. For details on extraction methods (AB1-6 and T1-2) refer to Figure 1.

A Principal Component (PC) Analysis (PCA) was performed to verify whether the different extraction methods impacted protein LC-MS quantitative data. A plot of PC1 (60.7% variance) against PC2 (32.9% variance) clearly separates urea-based methods from guanidine-HCl-based methods (Figure 2). 

Each of the six methods are well defined and do not cluster together. Extraction methods 3–6, which include an initial precipitation step, are further isolated.

Table 1 indicates the concentration of the protein extracts as well as the number of protein groups quantified in Genedata expressionist. 

Extraction method 1 yields the greatest protein concentrations: 6.6 mg/mL in apical buds and 3.5 mg/mL in trichomes, followed by extraction methods 2, 4, 6, 3 and 5. Overall, 571 proteins were quantified and the extraction methods recovering most intact proteins in apical buds are methods 2 (335 ± 15), 4 (314 ± 16) and 6 (264 ± 18). In our experiment, method 1 yielding the highest protein concentrations does not equate larger numbers of proteins resolved by LC-MS. Perhaps *C. sativa* proteins recovered by method 1 are not compatible with our downstream analytical techniques (LC-MS). In trichomes, the method yielding the highest number of intact proteins is extraction method 2 (249 ± 45). Extraction methods 2, 4 and 6 all conclude by a resuspension step in a guanidine-HCl buffer, which consequently is the buffer we recommend for intact protein analysis.

As far as we know, this is the first time a gel-free intact protein analysis is presented. In the literature, intact protein analysis on *C. sativa* has thus far been achieved using 2-DE [8,11,12], which resolved 800, 300 and 1102 spots, respectively. The old-fashioned technique 2-DE separates intact proteins based first on their isoelectric point and second on their molecular weight (MW). Because it is time-consuming, labour-intensive and of low throughput, 2-DE has now been superseded by liquid-based techniques, such as LC-MS. In the present study we have chosen to separate intact proteins of medicinal cannabis based on their hydrophobicity using RP-LC and a C8 stationary phase online with a high-resolution mass analyser which separates ionised intact proteins based on their mass-to-charge ratio (*m*/*z*). In our hands, this strategy has proven successful to resolve complex mixtures of intact proteins from different types of samples, such as milk [17,18,19], wheat flour and Buffalo grass leaves. 

### 2.2. Tryptic Peptides Analysis

The 25 tryptic digests of medicinal cannabis extracts and BSA sample were separated by nLC and analysed by ESI-MS/MS in duplicates. BSA was used as a control for the digestion with the mixture of endoproteases, trypsin and Lys-C, cleaving arginine (R) and lysine (K) residues. BSA was successfully identified with overall 88 peptides covering 75.1% of the total sequence (Supplementary Table S1), indicating that both protein digestions and nLC-MS/MS analyses were efficient. 

Nano LC-MS/MS profiles are very complex (Supplementary Figure S2) with altogether 105,249 LC-MS peaks (peptide ions) clustered into 43,972 isotopic clusters, with up to 11,540 MS/MS events. If we consider apical bud patterns only, it is visually evident that guanidine-HCl-based extraction methods (2, 4 and 6) generate a lot more peaks than urea-based methods (1, 3 and 5) (Supplementary Figure S2). Indeed, extraction methods 2, 4 and 6 resolve 23,952 (CV 18%), 55,443 (CV 7%) and 59,084 (CV 11%) nLC-MS peaks, respectively, while extraction methods 1, 3 and 5 yield 8484 (CV 25%), 18,884 (CV 9%) and 2537 (CV 22%) nLC-MS peaks, respectively (Supplementary Table S2). Reduced CVs also indicate greater reproducibility of guanidine-HCl-based extraction methods. As far as trichomes are concerned, extraction methods 1 and 2 yield comparable patterns, albeit with less nLC-MS peaks than those of apical buds (Supplementary Table S2).

**Figure 3:**
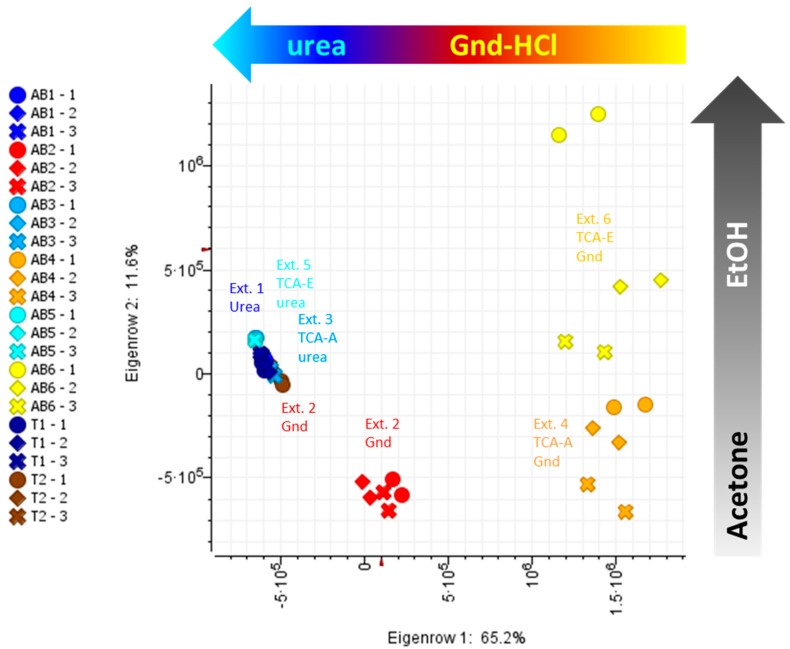
Principal component analysis (PCA) using bottom-up proteomics data. AB, apical buds; T, trichomes; 1–6, extraction methods 1 to 6. For details on extraction methods (AB1-6 and T1-2) refer to Figure 1.

The volumes of all the peaks comprised into a cluster were summed and the sum was used as a proxy for the amounts of the tryptic peptides. PCA were performed on the summed volumes of the 43,972 peptide clusters. A biplot of PC 1 against PC 2 illustrates the separation of guanidine-HCl based-methods from urea-based methods along PC 1 (65.2% variance), and the distinction between acetone (method 4) and ethanol (method 6) precipitations along PC 2 (11.6% variance) (Figure 3).

Table 2 indicates the number of peptides identified with high score (Xcorr > 1.5) by SEQUEST algorithm and matching one of the 590 AA sequences we retrieved from *C. sativa* and closely related species for the database search. 

Overall, 488 peptides were identified (Supplementary Table S3) and the extraction methods yielding the greatest number of database hits in apical buds were methods 4 (435 ± 9), 6 (429 ± 6) and 2 (356 ± 20). In trichomes, the method yielding the highest number of identified peptides was extraction method 2 (102 ± 23). Similar to our conclusions from intact protein analyses, we also recommend guanidine-HCl-based extraction methods (2, 4 and 6) for trypsin digestion followed by shotgun proteomics.

**Figure 4:**
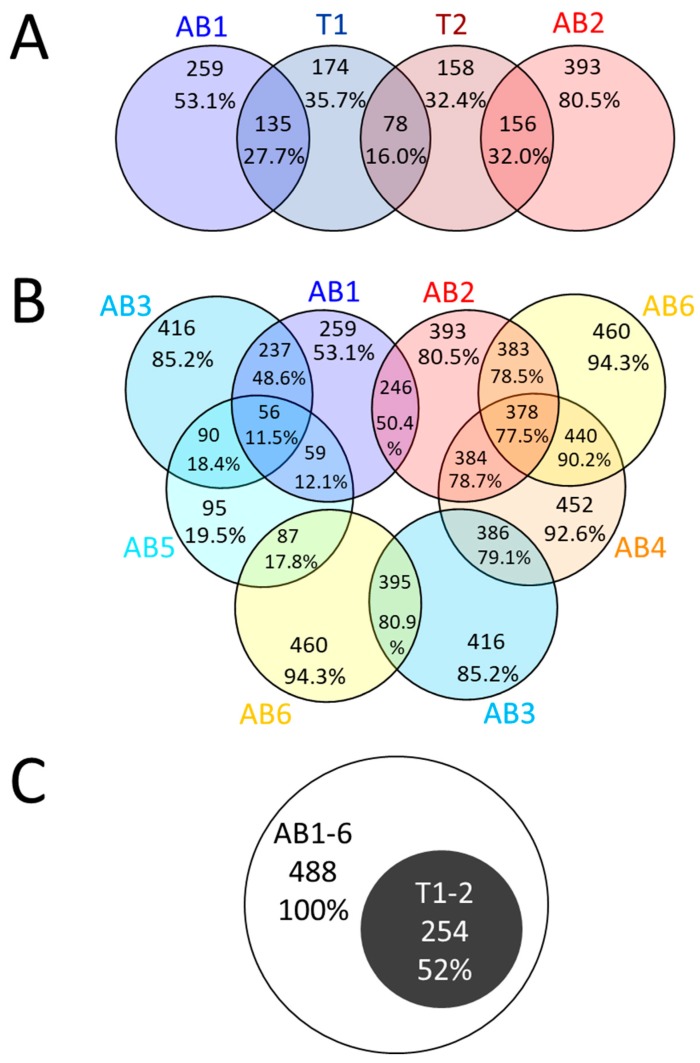
Venn diagram using the number of tryptic peptides identified by bottom-up proteomics. (**A**) Apical bud and extraction method 1 to 6; (**B**) trichomes and apical buds and extraction methods 1 and 2; and (**C**) all apical buds across all 2 methods and trichomes across both methods. For details on extraction methods 1–6 refer to Figure 1.

To further compare the extraction methods with each other, Venn diagrams were produced on the 488 identified peptides (Figure 4). 

If we start with the trichomes and compare the simplest methods, extraction methods 1 and 2 which only involve a single resuspension step of the frozen ground plant powder into a protein-friendly buffer, we observe similar identification success 35.7% (174 out of 488 peptides) for T1 and 32.4% (158 peptides) for T2, with little overlap (16.0%; 78 peptides) between the two (Figure 4A). Therefore, both methods are complementary. If we compare trichomes and apical buds, an overlap of 27.7% (135 peptides) is observed with extraction method 1 (urea-based buffer) while 32.0% (156 peptides) of database hits are shared between both tissues when extraction method 2 (guanidine-HCl) is employed (Figure 4A). Whilst both outcomes are comparable, we would thus advice employing method 2 when handling cannabis trichomes. If we now turn our attention to just apical buds, we can see that half of the identified peptides are common between methods 1 and 2 (AB1-AB2, 246 peptides; 50.4%). Guanidine-HCl-based methods (AB2, AB4 and AB6) share a majority of hits (77.5%; 378 peptides), whereas urea-based methods (AB1, AB3 and AB5) only share 11.5% (56) of identified peptides (Figure 4B). This indicates that guanidine-HCl-based methods not only yield more identified peptides but also more consistently. Interestingly, the two most different methods (AB3 and AB6 employing different precipitant solvents and different resuspension buffers) share 80.9% (395) of the identified peptides (Figure 4B), suggesting that the initial precipitation step would make the subsequent resuspension step more homogenous, irrespective of the buffer used. All the 254 peptides identified from trichomes were also identified in apical buds (Figure 4C). Therefore, in our hands, protein extraction from trichome did not yield unique protein identification. This might be explained by the fact that due to limited sample recovery only two extraction methods were tested on trichomes. A more refined method on trichomes might help enrich in metabolism unique to *C. sativa* species.

### 2.3. Proteins Identified by Bottom-Up Proteomics

Table 3 lists the 160 protein accessions from the 488 peptides (Supplementary Table S3) identified from cannabis mature apical buds and trichomes in this study. 

These 160 accessions correspond to 99 protein annotations (including 56 enzymes) and 15 pathways (Table 3). Most proteins (83.1%) matched a *C. sativa* accession, 5% of the accessions came from European hop and 11.8% of the accessions came from *Boehmeria nivea*, all of which annotated as small auxin upregulated (SAUR) proteins. 

**Figure 5:**
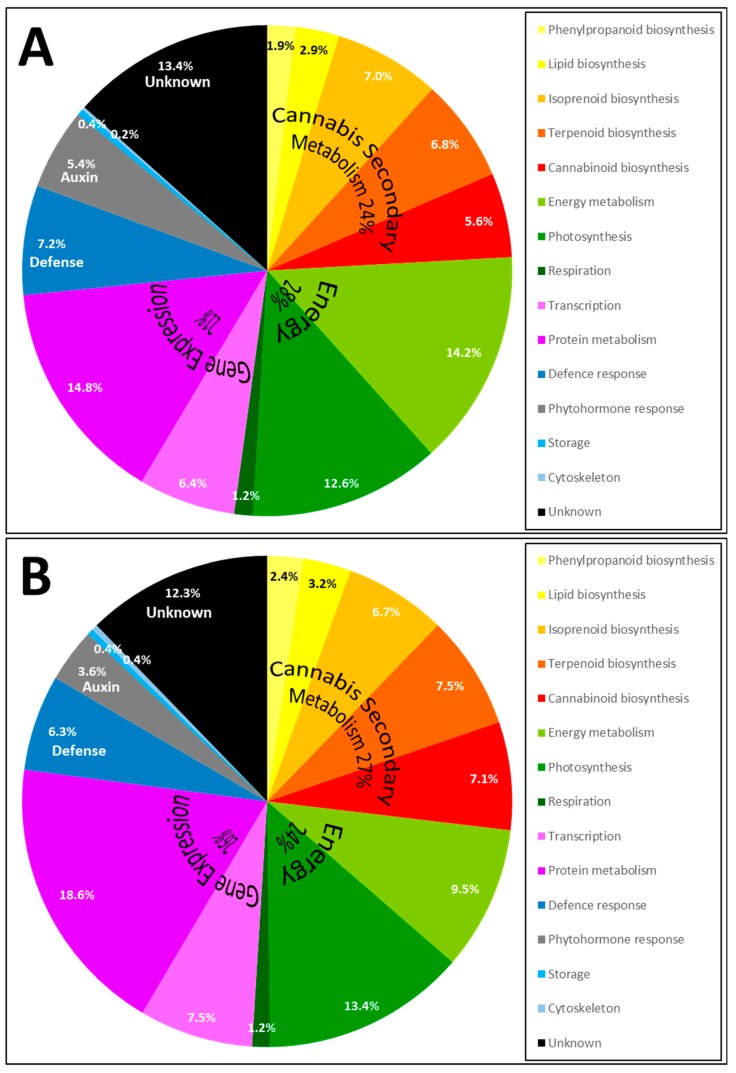
Pie chart of the pathways in which identified Cannabis proteins are involved in and recovered from apical buds (**A**) and trichomes (**B**).

The frequency of identified proteins for each pathway is illustrated in a pie chart (Figure 5). 

Most proteins belong to the cannabis secondary metabolism (24% in apical buds and 27% in trichomes), which encompasses the biosynthesis of phenylpropanoids, lipid, isoprenoids, terpenoids and cannabinoids [7]. Cannabinoid biosynthesis (5.6% in apical buds and 7.1% in trichomes) and terpenoid biosynthesis (6.8% in apical buds and 7.5% in trichomes) is a significant portion of this classification, with many terpene synthases (TPS, Table 3). A detailed analysis of terpene synthases and terpene metabolites in cannabis flowers was recently published [20]; nine cannabis terpene synthases (CsTPS) were discovered and their transcripts demonstrated to be upregulated in trichomes relative to nonresinous tissues. We have identified two major enzymes involved in monolignol biosynthesis: phenylalanine ammonia-lyase (PAL) and 4-coumarate:CoA ligase (4CL) (Table 3); with three accessions the phenylpropanoid pathway only contributes to 1.9% and 2.4% of the identification results, respectively in apical buds and trichomes (Figure 5). In a recent time course study on hemp hypocotyls, numerous enzymes involved in cellulose and lignin deposition were analysed using microscopic observations, lignin analysis, gel-based and shotgun proteomics, along with enzyme activity assays [9]; it would be of interest to repeat such an elaborate study on cannabis reproductive organs during the maturation process. The second most prominent category is energy metabolism (28% in apical buds and 24% in trichomes), comprising photosynthesis and respiration (Figure 5). The third major category is gene expression metabolism (22% in apical buds and 26% in trichomes) which includes transcriptional and translational mechanisms (Figure 5). A significant portion of protein accessions remain of unknown function (13.4% in apical buds and 12.3% in trichomes). To summarise, the pattern in the trichomes is very similar to that of apical buds albeit with an enrichment of cannabinoid biosynthetic proteins (7.1% in trichomes and 5.6% in apical buds) and terpenoid biosynthetic proteins (7.5% in trichomes and 6.8% in apical buds).

Most of the proteins identified in this study were also identified in a study on hempseeds published in 2016 [10], in which a labour-intensive strategy combined combinatorial peptide ligand libraries, SDS-PAGE separation, nLC-ESI-MS/MS identification, and database search including accessions from *C. sativa* and *Arabidopsis thaliana*. A total of 181 accessions were thereby identified, yet only 56 (31%) belonged to *C. sativa*, the rest matching *A. thaliana* accessions [10]. We have also searched all the *viridiplantea* accessions available from NCBI and consequently obtained many more hits (data not shown). Here, we only present identification results obtained with the Uniprot *C. sativa* list of sequences (590 AA sequences, including 440 sequences from *C. sativa*, 72 sequences from Chinese grass and 77 sequences from European hop) because our aim is to assess the efficiency of the six protein extraction methods by validating the occurrence of cannabis unique metabolisms, such as the phytocannabinoid pathway (presented in the next section). 

In a different study on hempseeds [11], proteins were resolved by 2-DE followed by MS/MS analysis resulting in the identification of 168 proteins mostly from rice, but only one protein (Ubiquitin-like protein SMT3) from *C. sativa*. This reveals that plant database have improved since 2012 and incorporated more accessions from *C. sativa*. Proteomics studies on *C. sativa* published prior to that year [8,12] did not produce a hit against *C. sativa* sequences, as databases hosted only a few *C. sativa* sequences. We have retrieved all the entries referenced under the keyword “*Cannabis sativa*” in UniprotKB and produced a histogram of their distribution per year of creation. Most entries (81%) were created in 2015–2017, with only 10 created in 2018 (Supplementary Figure S3). This explains why only studies published after 2015 could report database hits against *C. sativa* entries. Whilst ever-increasing, the number of sequences from *C. sativa* publicly available in Uniprot is far from sufficient, and the proteomics community still must rely on information from unrelated plants species, such as Arabidopsis, and rice, to identify cannabis proteins. A draft genome and transcriptome of *C. sativa* was released in 2011 [21]. After a lull of several years the same research group just released on 8 November 2018 a physical and genetic map of *C. sativa* which reveals extensive rearrangements at the THCA/CBDA synthase loci [22]. On 31 October 2018, another research group reported that CBDAS and THCAS gene clusters were associated with transposable elements [23]. Also, in November 2018, the Open Cannabis Project (OCP, https://opencannabisproject.org) has made a dataset of approximately 850 strains of cannabis available in BigQuery as part of the 1000 Cannabis Genomes Project. This should help boost the number of *C. sativa* protein accessions in public protein databases in the very near future.

### 2.4. Enzymes Involved in Phytocannabinoid Pathway

**Figure 6:**
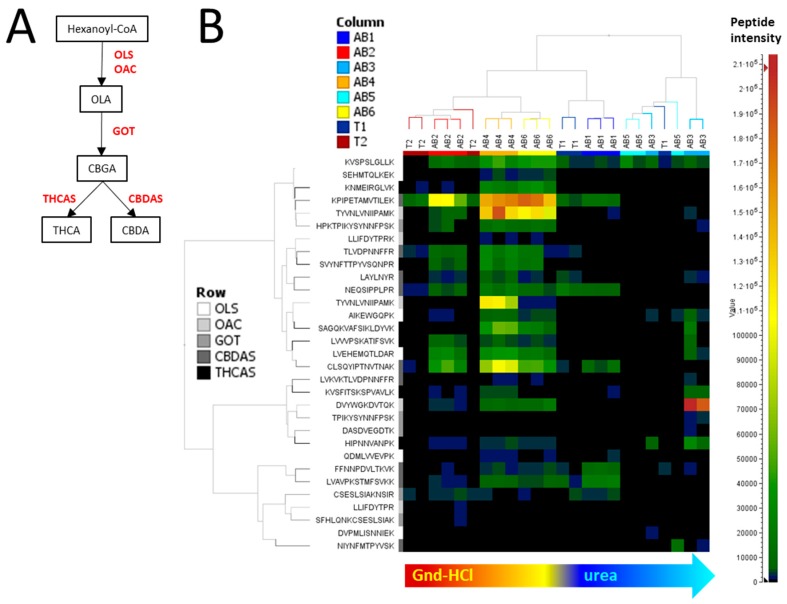
Impact of extraction methods on enzymes involved in cannabinoid biosynthesis identified by bottom-up proteomics. (**A**) Cannabinoid pathway. Enzymes in red were identified in this work. (**B**) Two-dimensional hierarchical clustering of enzymes involved in cannabinoid synthesis. Columns represent extraction method per tissue types (AB, apical bud; T, trichomes. See Column legend as well) and rows represent the peptides identified from enzymes of interest. Peptides from the same enzymes bear the same shade of grey (see Row legend as well). CBDA, Cannabidiolic acid; CBDAS, CBDA synthase; CBGA, Cannabigerolic acid; GOT, geranyl-pyrophosphate-olivetolic acid geranyltransferase; OAC, Olivetolic acid cyclase; OLA, Olivetolic acid; OLS, 3,5,7-trioxododecanoyl-CoA synthase; THCA, delta9-tetrahydrocannabinolic acid; THCAS, THCA synthase.

To validate the extraction methods, we focused on the cannabis-specific pathway that attracts most of the interest in the medicinal cannabis industry, namely the biosynthesis of phytocannabinoids [24]. In our bottom-up results, five enzymes involved in phytocannabinoid biosynthesis and whose functions were described in the introduction were identified: 3,5,7-trioxododecanoyl-CoA synthase (OLS) identified with seven peptides (19% coverage), olivetolic acid cyclase (OAC) identified with six peptides (13% coverage), geranyl-pyrophosphate-olivetolic acid geranyltransferase (GOT) identified with five peptides (17% coverage), delta9-tetrahydrocannabinolic acid synthase (THCAS) identified with six peptides (15% coverage) and cannabidiolic acid synthase (CBDAS) identified with eight peptides (17% coverage). The steps these enzymes catalyse are summarised in Figure 6A. 

The two-dimensional hierarchical clustering analysis (2-D HCA) presented in Figure 6B clusters guanidine-HCl-based samples away from the urea-based samples, in particular methods 3 and 5. Peptides do not cluster based on the protein they belong to. Extraction methods 4 and 6 allowed the detection and quantitation of most of the 31 peptides belonging to these phytocannabinoid enzymes, with respectively 87% and 86% of the peptides being up-regulated (Figure 6B). Both methods apply a TCA/solvent precipitation step followed by resuspension in a guanidine-HCl buffer. Consequently, this is the protein extraction method we recommend in order to recover and analyse the phytocannabinoid-related enzymes using a bottom-up proteomics strategy.

As more genomes are released, the identification of additional genes in the biosynthetic pathways is likely. Already THCAS and CBDAS gene clusters have been identified where the genes are highly homologous [22,23]. The function of all these genes is yet to be confirmed and proteomics methods will be useful to identify which of genes are translated at high efficiency in different cannabis strains. In designing medicinal cannabis strains for specific therapeutic requirements, either by genomic assisted breeding techniques (especially genomic selection) or through genome editing this protein expression information will be critical to optimise cannabinoid and terpene biosynthesis.

## 3. Materials and Methods 

The experimental design is schematised in Figure 1.

### 3.1. Plant Materials

#### 3.1.1. Apical Bud Sampling and Grinding

Fresh plant material was obtained from the Victorian Government Medicinal Cannabis Cultivation Facility. The top three centimetres of the apical bud was excised using secateurs, placed into a labelled paper bag, snap frozen in liquid nitrogen and stored at −80 °C until grinding. Samples were collected in triplicates. Frozen buds were ground in liquid nitrogen using a mortar and pestle. The ground frozen powder was transferred into a 15 mL tube and stored at stored at −80°C until protein extraction.

#### 3.1.2. Trichome Recovery

The top three centimetres of the apical bud was cut using secateurs and placed into a labelled paper bag. Samples were collected in triplicates. Trichome recovery was inspired by the procedure devised by Yerger and colleagues [25], with modifications. The bud was further partially trimmed with the secateurs into smaller pieces and placed into a 50 mL tube. Approximately 10 mL liquid nitrogen was added to the tube and the cap was loosely attached. The tube was then vortexed for 1 min while covering the loosened lid with one hand to avoid spilling the plant material. The cap was removed, and the content of the tube was discarded by inverting the tube and tapping it on the bench, while the trichomes stuck to the walls of the tube. The process was repeated in the same tube until all the apical bud was trimmed. Tubes were stored at −80 °C until protein extraction.

### 3.2. Protein Extraction Methods

For the apical bud extraction, one 50 mg scoop of ground frozen powder was transferred into a 2 mL microtube kept on ice prefilled with 1.8 mL precipitant or 0.5 mL resuspension buffer depending on the extraction method employed (described below and in Figure 1). All six extraction methods described hereafter were applied to the apical bud samples. For the trichome extraction, all trichomes stuck to the walls of the tubes were resuspended into the solutions and volumes specified below. Due the limited amount of trichomes recovered, only extraction methods 1 and 2 were attempted.

#### 3.2.1. Extraction 1: Resuspension in Urea Buffer

Plant material was resuspended in 0.5 mL of urea buffer (6M urea, 10mM DTT, 10mM Tris-HCl pH 8.0, 75mM NaCl, and 0.05% SDS). The tubes were vortexed for 1 min, sonicated for 5 min, vortexed again for 1 min. The tubes were centrifuged for 10 min at 13,500 rpm. The supernatant was transferred into fresh 1.5 mL tubes and stored at −80 °C until protein assay.

#### 3.2.2. Extraction 2: Resuspension in Guanidine Hydrochloride Buffer

Plant material was resuspended in 0.5 mL of guanidine-HCl buffer (6 M guanidine-HCl, 10 mM DTT, 5.37 mM sodium citrate tribasic dihydrate and 0.1 M Bis-Tris). The tubes were vortexed for 1 min, sonicated for 5 min, vortexed again for 1 min. The tubes were centrifuged for 10 min at 13,500 rpm and at 4 °C. The supernatant was transferred into fresh 1.5 mL tubes and stored at −80 °C until protein assay.

#### 3.2.3. Extraction 3: TCA/Acetone Precipitation Followed by Resuspension in Urea Buffer

Plant material was resuspended in 1.8 mL ice-cold 10% TCA/10mM DTT/acetone (*w*/*w*/*v*) by vortexing for 1 min. Tubes were left at −20 °C overnight. The next day, tubes were centrifuged for 10 min at 13,500 rpm and at 4 °C. The supernatant was removed, and the pellet was resuspended in ice-cold 10mM DTT/acetone (*w*/*v*) by vortexing for 1 min. Tubes were left at −20 °C for 2 h. The tubes were centrifuged as specified before and the supernatant removed. This washing step of the pellet was repeated once more. The pellets were dried for 30 min under a fume hood. The dry pellet resuspended in 0.5 mL of urea buffer as described in Extraction 1. 

#### 3.2.4. Extraction 4: TCA/Acetone Precipitation Followed by Resuspension in Guanidine Hydrochloride Buffer

Plant material was processed as detailed in Extraction 3, except that the dry pellet was resuspended in 0.5 mL of guanidine-HCl buffer.

#### 3.2.5. Extraction 5: TCA/Ethanol Precipitation Followed by Resuspension in Urea Buffer

Plant material was processed as detailed in Extraction 3, except that acetone was replaced with ethanol.

#### 3.2.6. Extraction 6: TCA/Ethanol Precipitation Followed by Resuspension in Guanidine Hydrochloride Buffer

Plant material was processed as detailed in Extraction 4, except that acetone was replaced with ethanol.

### 3.3. Alkylation and Protein Assay

Following protein extraction, DTT-reduced proteins were alkylated by adding 10 µL of 1M iodoacetamide (IAA)/water (w/v) solution to the extracts to reach a 20 mM final IAA concentration. The tubes were vortexed for 1 min and left to incubate at room temperature in the dark for 60 min.

Protein extracts from apical buds were diluted ten times into their respective resuspension buffer and protein extracts from trichomes were diluted four times. The protein concentrations were measured in triplicates using the Microplate BCA protein assay kit (Pierce) following the manufacturer’s instructions. Bovine Serum Albumin (BSA) from the kit was used as a standard as per instructions. 

### 3.4. Trypsin/LysC protein Digestion and Desalting

#### 3.4.1. Protease Digestion

An aliquot corresponding to 100 μg of plant proteins was used for protein digestion as follows. The DTT-reduced and IAA-alkylated proteins were diluted six times using 50 mM Tris-HCl pH 8 to drop the resuspension buffer molarity below 1 M. Trypsin/Lys-C protease (Mass Spectrometry Grade, 100 μg, Promega) was carefully solubilised in 1 mL of 50 mM Tris-HCl pH 8. A 40 µL aliquot of trypsin/Lys-C solution was added and gently mixed with the plant extracts thus achieving a 1:25 ratio of protease:plant proteins. The mixture was left to incubate overnight (19 h) at 37 °C in the dark. The digestion reaction was stopped by lowering the pH of the mixture using a 10% formic acid (FA) in H_2_O (*v*/*v*) to a final concentration of 1% FA.

BSA was also digested under the same conditions to be used as a control for digestion and nLC-MS/MS analysis.

#### 3.4.2. Desalting

The 25 tryptic digests were desalted using solid phase extraction (SPE) cartridges (Sep-Pak C18 1cc Vac Cartridge, 50 mg sorbent, 55–105 μm particle size, 1 mL, Waters) by gravity as described in [26].

A 90 µL aliquot of peptide digest was mixed with 10 µL 1ng/μL Glu-Fibrinopeptide B (Sigma), as an internal standard. The peptide/internal standard mixture was transferred into a 100 μL glass insert placed into a glass vial. The vials were positioned into the autosampler at 4 °C for immediate analyses by nLC-MS/MS.

### 3.5. Intact Protein Analysis by Ultraperformance Liquid Chromatography Mass Spectrometry

#### 3.5.1. UPLC separation

The Ultraperformance Liquid Chromatography Mass Spectrometry (UPLC-MS) analyses of the 24 plant protein extracts were performed in duplicates for a total of 48 MS files. Protein extracts were chromatographically separated using a UHPLC 1290 Infinity Binary LC system (Agilent) and an Aeris™ WIDEPORE XB-C8 column (3.6 µm, 200 Å, 150 × 2.1 mm, Phenomenex) kept at 75 °C as described in [17,18,19]. Flow rate was 0.2 mL/min. Mobile phase A contained 0.1% FA in water and mobile phase B contained 0.1% FA in acetonitrile. UPLC gradient was as follows: starting conditions 3% B, held for 2.5 min, ramping to 60% B in 27.5 min, ramping to 99% B in 1 min and held at 99% B for 4 min, lowering to 3% B in 0.1 min, equilibration at 3% B for 4.9 min. A 10 µL injection volume was applied to each protein extract, irrespective of their protein concentration. Each extract was injected twice.

#### 3.5.2. MS acquisition

During the 40 min chromatographic separation, plant intact proteins were analysed using an Orbitrap Velos hybrid ion trap-Orbitrap mass spectrometer (ThermoFisher Scientific) online with the UPLC and fitted with a heated electrospray ionisation (HESI) source. HESI parameters were as follows: capillary heated to 300 °C, source heated to 250 °C, sheath gas flow 30, auxiliary gas flow 10, sweep gas flow 2, 3.6 kV, 100 µA and S-Lens RF level 60%. SID was set at 15V.

For the first 2.5 min, LC flow was sent to waste, switched to source from 2.5 to 38 min, and finally switched back to waste for the last minute of the 40 min run. Spectra were acquired in positive ion mode using the full MS scan mode of the Fourier Transform (FT) orbitrap mass analyser at a resolution of 60,000 using a 500–2000 *m*/*z* mass window and 6 microscans to improve signal-to-noise ratio. FT Penning gauge pressure difference was set at 0.05 E-10 Torr to improve the signal intensity of large molecules such as intact proteins.

All LC-MS files are available from the stable public repository MassIVE at the following URL: http://massive.ucsd.edu/ProteoSAFe/datasets.jsp with the accession number MSV000083191.

### 3.6. Peptide Digest Analysis by Nanoliquid Chromatography-Tandem Mass Spectrometry 

The Nanoliquid Chromatography-Tandem Mass Spectrometry (nLC-ESI-MS/MS) analyses were performed on the 25 peptide digests in duplicates thus yielding 50 MS/MS files. Chromatographic separation of the peptides was performed by reverse phase (RP) using an Ultimate 3000 RSLCnano System (Dionex) online with an Orbitrap Velos hybrid ion trap-Orbitrap mass spectrometer (ThermoFisher Scientific). The parameters for nLC and MS/MS are described in [26] and here. A 1 μL aliquot (0.1 μg peptide) was loaded using a full loop injection mode onto a trap column (Acclaim PepMap100, 75 μm x 2 cm, C18 3 μm 100 Å, Dionex) at a 3 μL/min flow rate and switched onto a separation column (Acclaim PepMap100, 75 μm x 15 cm, C18 2 μm 100 Å, Dionex) at a 0.4 μL/min flow rate after 3 min. The column oven was set at 30 °C. Mobile phases for chromatographic elution were 0.1% FA in H_2_O (*v*/*v*) (phase A) and 0.1% FA in ACN (*v*/*v*) (phase B). Ultraviolet (UV) trace was recorded at 215 nm for the whole duration of the nLC run. A linear gradient from 3% to 40% of ACN in 35 min was applied. Then ACN content was brought to 90% in 2 min and held constant for 5 min to wash the separation column. Finally, the ACN concentration was lowered to 3% over 0.1 min and the column reequilibrated for 5 min. On-line with the nLC system, peptides were analysed using an Orbitrap Velos hybrid ion trap-Orbitrap mass spectrometer (Thermo Scientific). Ionization was carried out in the positive ion mode using a nanospray source. The electrospray voltage was set at 2.2 kV and the heated capillary was set at 280 °C. Full MS scans were acquired in the Orbitrap Fourier Transform (FT) mass analyser over a mass range of 300 to 2000 *m*/*z* with a 60,000 resolution in profile mode. MS/MS spectra were acquired in data-dependent mode. The 20 most intense peaks with charge state ≥ 2 and a minimum signal threshold of 10,000 were fragmented in the linear ion trap using collision-induced dissociation (CID) with a normalised collision energy of 35%, 0.25 activation Q and activation time of 10 msec. The precursor isolation width was 2 *m*/*z*. Dynamic exclusion was enabled, and peaks selected for fragmentation more than once within 10 sec were excluded from selection for 30 sec. Each digest was injected twice. Blanks (1 μL of mobile phase A) were injected in between each set of six extraction replicates and analysed over a 20 min nLC run to minimise carry-over.

### 3.7. Database Search for Protein Identification

Database searching of the 50 MS .RAW files was performed in Proteome Discoverer (PD) 1.4 using SEQUEST algorithm. All 589 *C. sativa* protein sequences publicly available on 13 December 2018 from UniprotKB (www.uniprot.org; key word used “*Cannabis sativa*”) were downloaded as a FASTA file. These also included 77 sequences from the European hop *Humulus lupulus*, the closest relative to *C. sativa* [27], as well as 72 sequences from the Chinese grass *Boehmeria nivea*, also closely related to cannabis [27]. Because GOT sequence was not included, we retrieved it from patent WO 2011/017798 Al [28] and included it to the FASTA file (590 entries). The FASTA file was imported and indexed in PD 1.4. The SEQUEST algorithm was used to search the indexed FASTA file. The database searching parameters specified trypsin as the digestion enzyme and allowed for up to two missed cleavages. The precursor mass tolerance was set at 10 ppm, and fragment mass tolerance set at 0.5 Da. The peptide absolute Xcorr threshold was set at 0.4 and protein relevance threshold was set at 1.5. Carbamidomethylation (C) was set as a static modification. Oxidation (M), phosphorylation (STY), conversion from Gln to pyro-Glu (N-term Q) and Glu to pyro-Glu (N-term E), and deamination (NQ) were set as dynamic modifications. The target decoy peptide-spectrum match (PSM) validator was used to estimate false discovery rates (FDR). At the peptide level, peptide confidence value set at high was used to filter the peptide identification, and the corresponding FDR on peptide level was less than 1%. At the protein level, protein grouping was enabled.

All nLC-MS/MS files are available from the stable public repository MassIVE at the following URL: http://massive.ucsd.edu/ProteoSAFe/datasets.jsp with the accession number MSV000083191.

### 3.8. Data Processing and Statistical Analyses

#### 3.8.1. LC-MS and nLC-MS/MS Data Processing

The data files obtained following UPLC-MS analysis were processed in the Refiner MS module of Genedata Expressionist^®^ 11.0 with the following parameters (1) Rentention Time (RT) Structure Removal using a 5 scan minimum RT length; (2) *m*/*z* Structure Removal using 8 points minimum *m*/*z* length; (3) Chromatogram Chemical Noise Reduction using 7 scan smoothing and a moving average estimator; (4) Spectrum Smoothing using a Savitzky-Golay algorithm with 5 points *m*/*z* window and a polynomial order of 3; (5) Chromatogram RT Alignment using a pairwise alignment-based tree and 50 RT scan search interval; (6) Chromatogram Peak Detection using a 0.3 min minimum peak size, 0.02 Da maximum merge distance, a boundaries merge strategy, a 30% gap–peak ratio, a curvature-based algorithm, using both local maximum and inflection points to determine boundaries; (7) Chromatogram Isotope Clustering using a 4 scan RT tolerance, a 20 ppm *m*/*z* tolerance, a peptide isotope shaping method with protonation, charges from 2-25, mono-isotopic masses and variable charge dependency; (8) Singleton Filter; (9) Charge and Adduct Grouping (i.e., deconvolution) using a 50 ppm mass tolerance, a 0.1 min RT tolerance, a dynamic adduct list containing ions (H), and neutrals (-H_2_O, K-H, and Na-H); and (10) Export Analyst using group volumes.

The data files obtained following nLC-MS/MS analysis were processed in the Refiner MS module of Genedata Expressionist^®^ 11.0 with the following parameters (1) RT Structure Removal applying a minimum of 4 scans; (2) *m*/*z* Structure Removal applying a minimum of 8 points, (3) Chromatogram Chemical Noise Reduction using 5 scan smoothing, a moving average estimator, a 25 scan RT window, a 30% quantile and clipping an intensity of 20; (4) Grid using an adaptive grid with 10 scans and 10% deltaRT smoothing; (5) Chromatogram RT Alignment using a pairwise alignment-based tree and 50 RT scan search interval; (6) Chromatogram Peak Detection using a 0.1 min minimum peak size, 0.03 Da maximum merge distance, a boundaries merge strategy, a 20% gap–peak ratio, a curvature-based algorithm, intensity-weighed and using inflection points to determine boundaries; (7) Chromatogram Isotope Clustering using a 0.3 min RT tolerance, a 0.1 Da *m*/*z* tolerance, a peptide isotope shaping method with protonation, charges from 2–6 and mono-isotopic masses; (8) Singleton Filter; (9) MS/MS Consolidation; (10) Proteome Discoverer Import using a Xcorr above 1.5; (11) Peak Annotation; and (12) Export Analyst using cluster volumes.

#### 3.8.2. Statistical Analyses

Statistical analyses were performed using the Analyst module of Genedata Expressionist^®^ 11.0 where columns denote plant samples and rows denote intact proteins or tryptic digest peptides. Principal Component Analyses (PCA) were performed on rows using a covariance matrix with 50% valid values and row mean as imputation. Two-dimensional hierarchical clustering (2-D HCA) was performed on both columns and rows using positive correlation and Ward linkage method. Venn diagrams were produced by exporting quantitative data of the identified peptides to Microsoft excel 2016 (office 365) spreadsheet and using the Excel function COUNT to establish the frequency of the peptides in the samples and across extraction methods. Venn diagrams were drawn in Microsoft Powerpoint 2016 (Office 365).

## 4. Conclusions

Six different extraction methods were assessed to analyse proteins from medicinal cannabis apical buds and trichomes. This is the first time protein extraction is optimised from cannabis reproductive organs, and the guanidine-HCl buffer employed here has never been used before on *C. sativa* samples. If we convert the number of intact proteins quantified and the number of peptides identified into percent and plot them as a histogram (Supplementary Figure S4), it is evident that guanidine-HCl-based methods (2, 4 and 6) are best suited to recover proteins from medicinal cannabis buds and preceding this with a precipitation step in TCA/acetone (AB4, method 4) or TCA/ethanol (AB6, method 6), ensures optimum trypsin digestion followed by MS/MS.

## Figures and Tables

**Table 1:** Protein extraction efficiency of the six methods tested on cannabis apical buds and trichomes assessed by top-down proteomics.

Tissue	Extraction Number	Extraction Method	Extraction Code	Protein Concentration (mg/mL)	Number of Proteins		
				Average (SD ^1^)	Average (SD ^1^)	CV ^2^	Percent
apical bud	extraction 1	Urea	AB1	6.58 (± 0.89)	254 (± 12)	4.80	44.51
apical bud	extraction 2	Gnd-HCl	AB2	3.50 (± 0.99)	335 (± 15)	4.47	58.58
apical bud	extraction 3	TCA-A/urea	AB3	0.63 (± 0.15)	247 (± 21)	8.69	43.23
apical bud	extraction 4	TCA-A/Gnd-HCl	AB4	1.50 (± 0.28)	314 (± 16)	5.13	54.90
apical bud	extraction 5	TCA-E/urea	AB5	0.60 (± 0.11)	201 (± 5)	2.64	35.11
apical bud	extraction 6	TCA-E/Gnd-HCl	AB6	0.76 (± 0.48)	264 (± 18)	6.84	46.18
trychome	extraction 1	Urea	T1	3.67 (± 0.39)	170 (± 5)	2.97	29.83
trychome	extraction 2	Gnd-HCl	T2	2.28 (± 1.17)	249 (± 45)	18.12	43.61
TOTAL					571		

^1.^ SD, standard deviation; ^2^ CV, coefficient of variation.

**Table 2:** Number of peptides identified by bottom-up proteomics with Xcorr >1.5.

Tissue	Extraction Number	Extraction Method	Extraction Code	Number of Hits		
				Average (SD ^1^)	CV ^2^	Percent
apical bud	extraction 1	Urea	AB1	211 (± 34)	16.09	43.24
apical bud	extraction 2	Gnd-HCl	AB2	356 (± 20)	5.51	72.88
apical bud	extraction 3	TCA-A/urea	AB3	265 (± 55)	20.70	54.23
apical bud	extraction 4	TCA-A/Gnd-HCl	AB4	435 (± 9)	2.09	89.07
apical bud	extraction 5	TCA-E/urea	AB5	41 (± 15)	35.71	8.33
apical bud	extraction 6	TCA-E/Gnd-HCl	AB6	429 (± 6)	1.33	87.91
trychome	extraction 1	Urea	T1	97 (± 22)	22.27	19.88
trychome	extraction 2	Gnd-HCl	T2	102 (± 23)	22.78	20.83
TOTAL				488		

^1.^ SD, standard deviation; ^2^ CV, coefficient of variation.

**Table 3:** List of proteins identified in medicinal cannabis apical buds and trichomes by bottom-up proteomics.

Protein Annotation	Abbreviation	Uniprot Accession or Patent Number	Species	Length (AA)	Number of Peptides Identified	EC Number	Function	Pathway
Small auxin upregulated protein	SAUR03	A0A172J1 × 8	*Boehmeria nivea*	93	1		response to auxin	Phytohormone response
Small auxin upregulated protein	SAUR20	A0A172J1Z7	*Boehmeria nivea*	147	1		response to auxin	Phytohormone response
Small auxin upregulated protein	SAUR23	A0A172J212	*Boehmeria nivea*	99	1		response to auxin	Phytohormone response
Small auxin upregulated protein	SAUR24	A0A172J211	*Boehmeria nivea*	102	1		response to auxin	Phytohormone response
Small auxin upregulated protein	SAUR28	A0A172J206	*Boehmeria nivea*	108	1		response to auxin	Phytohormone response
Small auxin upregulated protein	SAUR30	A0A172J210	*Boehmeria nivea*	100	1		response to auxin	Phytohormone response
Small auxin upregulated protein	SAUR31	A0A172J276	*Boehmeria nivea*	152	1		response to auxin	Phytohormone response
Small auxin upregulated protein	SAUR40	A0A172J219	*Boehmeria nivea*	105	1		response to auxin	Phytohormone response
Small auxin upregulated protein	SAUR44	A0A172J227	*Boehmeria nivea*	152	4		response to auxin	Phytohormone response
Small auxin upregulated protein	SAUR48	A0A172J226	*Boehmeria nivea*	133	1		response to auxin	Phytohormone response
Small auxin upregulated protein	SAUR54	A0A172J237	*Boehmeria nivea*	118	5		response to auxin	Phytohormone response
Small auxin upregulated protein	SAUR55	A0A172J229	*Boehmeria nivea*	97	3		response to auxin	Phytohormone response
Small auxin upregulated protein	SAUR58	A0A172J236	*Boehmeria nivea*	97	1		response to auxin	Phytohormone response
Small auxin upregulated protein	SAUR59	A0A172J243	*Boehmeria nivea*	106	5		response to auxin	Phytohormone response
Small auxin upregulated protein	SAUR60	A0A172J238	*Boehmeria nivea*	105	1		response to auxin	Phytohormone response
Small auxin upregulated protein	SAUR70	A0A172J249	*Boehmeria nivea*	183	1		response to auxin	Phytohormone response
Small auxin upregulated protein	SAUR71	A0A172J2A4	*Boehmeria nivea*	183	1		response to auxin	Phytohormone response
Small auxin upregulated protein	SAUR51	A0A172J290	*Boehmeria nivea*	97	1		response to auxin	Phytohormone response
Small auxin upregulated protein	SAUR52	A0A172J241	*Boehmeria nivea*	149	1		response to auxin	Phytohormone response
Cannabidiolic acid synthase	CBDAS	A6P6V9	*Cannabis sativa*	544	8	1.21.3.8	oxidative cyclisation of CBGA, producing CBDA	Cannabinoid biosynthesis
Geranylpyrophosphate:olivetolate geranyltransferase	GOT	WO 2011/017798 Al	*Cannabis sativa*	395	4		alkylation of OLA with geranyldiphosphate to form CBGA	Cannabinoid biosynthesis
Olivetolic acid cyclase	OAC	I1V0C9	*Cannabis sativa*	545	1	4.4.1.26	functions in concert with OLS/TKS to form OLA	Cannabinoid biosynthesis
Olivetolic acid cyclase	OAC	I6WU39	*Cannabis sativa*	101	5	4.4.1.26	functions in concert with OLS/TKS to form OLA	Cannabinoid biosynthesis
3,5,7-trioxododecanoyl-CoA synthase	OLS	B1Q2B6	*Cannabis sativa*	385	7	2.3.1.206	olivetol biosynthesis	Cannabinoid biosynthesis
Tetrahydrocannabinolic acid synthase	THCAS	A0A0H3UZT7	*Cannabis sativa*	325	1	1.21.3.7	oxidative cyclisation of CBGA, producing THCA	Cannabinoid biosynthesis
Tetrahydrocannabinolic acid synthase	THCAS	Q33DP7	*Cannabis sativa*	545	1	1.21.3.7	oxidative cyclisation of CBGA, producing THCA	Cannabinoid biosynthesis
Tetrahydrocannabinolic acid synthase	THCAS	Q8GTB6	*Cannabis sativa*	545	4	1.21.3.7	oxidative cyclisation of CBGA, producing THCA	Cannabinoid biosynthesis
Putative kinesin heavy chain	kin	Q5TIP9	*Cannabis sativa*	145	1		microtubule-based movement	Cytoskeleton
Betv1-like protein	Betv1	I6XT51	*Cannabis sativa*	161	38			Defence response
ATP synthase subunit alpha	atp1	A0A0M5M1Z3	*Cannabis sativa*	509	12		Produces ATP from ADP	Energy metabolism
ATP synthase subunit alpha	atp1	E5DK51	*Cannabis sativa*	349	1		Produces ATP from ADP	Energy metabolism
ATP synthase subunit 4	atp4	A0A0M4S8F3	*Cannabis sativa*	198	7		Produces ATP from ADP	Energy metabolism
ATP synthase subunit alpha	atpA	A0A0C5ARX6	*Cannabis sativa*	507	9		Produces ATP from ADP	Energy metabolism
ATP synthase subunit beta	atpB	F8TR83	*Cannabis sativa*	413	1	3.6.3.14	Produces ATP from ADP	Energy metabolism
ATP synthase CF1 epsilon subunit	atpE	A0A0C5AUH9	*Cannabis sativa*	133	1		Produces ATP from ADP	Energy metabolism
ATP synthase subunit beta, chloroplastic	atpF	A0A0C5AUE9	*Cannabis sativa*	189	2		Component of the F(0) channel	Energy metabolism
NADH-ubiquinone oxidoreductase chain 1	nad1	A0A0M4S8G1	*Cannabis sativa*	324	1	7.1.1.2		Energy metabolism
NADH-ubiquinone oxidoreductase chain 5	nad5	A0A0M4RVP1	*Cannabis sativa*	669	1	7.1.1.2		Energy metabolism
NADH dehydrogenase subunit 7	nad7	A0A0M4S7M8	*Cannabis sativa*	394	1	7.1.1.2		Energy metabolism
NADH dehydrogenase subunit 9	nad9	A0A0M4R4N3	*Cannabis sativa*	190	2	7.1.1.2		Energy metabolism
NADH dehydrogenase subunit 7	nadhd7	A0A0X8GLG5	*Cannabis sativa*	394	1			Energy metabolism
NADH-quinone oxidoreductase subunit H	ndhA	A0A0C5APZ2	*Cannabis sativa*	363	1	1.6.5.11	NDH-1 shuttles electrons from NADH to quinones	Energy metabolism
NADH-quinone oxidoreductase subunit N	ndhB	A0A0C5B2K5	*Cannabis sativa*	510	1	1.6.5.11	NDH-1 shuttles electrons from NADH to quinones	Energy metabolism
NADH-quinone oxidoreductase subunit K	ndhE	A0A0C5AUJ8	*Cannabis sativa*	101	4	1.6.5.11	NDH-1 shuttles electrons from NADH to quinones	Energy metabolism
NADH-quinone oxidoreductase subunit C	ndhJ	A0A0C5B2I2	*Cannabis sativa*	158	2	1.6.5.11	NDH-1 shuttles electrons from NADH to quinones	Energy metabolism
1-deoxy-D-xylulose-5-phosphate reductoisomerase	DXR	A0A1V0QSG8	*Cannabis sativa*	472	2		Converts 2-C-methyl-D-erythritol 4P into 1-deoxy-D-xylulose 5P	Isoprenoid biosynthesis
Transferase FPPS1	FPPS1	A0A1V0QSH0	*Cannabis sativa*	341	1			Isoprenoid biosynthesis
Transferase FPPS2	FPPS2	A0A1V0QSH7	*Cannabis sativa*	340	3			Isoprenoid biosynthesis
Transferase GPPS large subunit	GPPS	A0A1V0QSH4	*Cannabis sativa*	393	2			Isoprenoid biosynthesis
Transferase GPPS small subunit	GPPS	A0A1V0QSG9	*Cannabis sativa*	326	1			Isoprenoid biosynthesis
Transferase GPPS small subunit2	GPPS	A0A1V0QSI1	*Cannabis sativa*	278	1			Isoprenoid biosynthesis
4-hydroxy-3-methylbut-2-en-1-yl diphosphate reductase	HDR	A0A1V0QSH9	*Cannabis sativa*	408	6		Converts (E)-4-hydroxy-3-methylbut-2-en-1-yl-2P into isopentenyl-2P	Isoprenoid biosynthesis
Isopentenyl-diphosphate delta-isomerase	IDI	A0A1V0QSG5	*Cannabis sativa*	304	7		Converts isopentenyl diphosphate into dimethylallyl diphosphate	Isoprenoid biosynthesis
Mevalonate kinase	MK	A0A1V0QSI0	*Cannabis sativa*	416	3	2.7.1.36	Converts (R)-mevalonate into (R)-5-phosphomevalonate	Isoprenoid biosynthesis
Diphosphomevalonate decarboxylase	MPDC	A0A1V0QSG4	*Cannabis sativa*	455	4			Isoprenoid biosynthesis
Phosphomevalonate kinase	PMK	A0A1V0QSH8	*Cannabis sativa*	486	4		Converts (R)-5-phosphomevalonate into (R)-5-diphosphomevalonate	Isoprenoid biosynthesis
Non-specific lipid-transfer protein	ltp	P86838	*Cannabis sativa*	20	3		transfer lipids across membranes	Lipid biosynthesis
Non-specific lipid-transfer protein	ltp	W0U0V5	*Cannabis sativa*	91	9		transfer lipids across membranes	Lipid biosynthesis
4-coumarate:CoA ligase	4CL	A0A142EGJ1	*Cannabis sativa*	544	1	6.2.1.12	forms 4-coumaroyl-CoA from 4-coumarate	Phenylpropanoid biosynthesis
4-coumarate:CoA ligase	4CL	V5KXG5	*Cannabis sativa*	550	3	6.2.1.12	forms 4-coumaroyl-CoA from 4-coumarate	Phenylpropanoid biosynthesis
Phenylalanine ammonia-lyase	PAL	V5KWZ6	*Cannabis sativa*	707	4	4.3.1.24	Catalyses L-phenylalanine = trans-cinnamate + ammonia	Phenylpropanoid biosynthesis
NAD(P)H-quinone oxidoreductase subunit 5, chloroplastic	ndhF	A0A0C5AUJ6	*Cannabis sativa*	755	1	1.6.5.-	NDH shuttles electrons from NAD(P)H:plastoquinone to quinones	Photosynthesis
Photosystem I P700 chlorophyll a apoprotein A1	pasA	A0A0U2DTB0	*Cannabis sativa*	750	2	1.97.1.12	bind P700, the primary electron donor of PSI	Photosynthesis
Photosystem I P700 chlorophyll a apoprotein A2	psaB	A0A0C5APY0	*Cannabis sativa*	734	2	1.97.1.12	bind P700, the primary electron donor of PSI	Photosynthesis
Photosystem I iron-sulfur center	psaC	A0A0C5AS17	*Cannabis sativa*	81	10	1.97.1.12	assembly of the PSI complex	Photosynthesis
Photosystem II CP47 reaction center protein	psbB	A9XV91	*Cannabis sativa*	488	1		binds chlorophyll in PSII	Photosynthesis
Ribulose bisphosphate carboxylase large chain	rbcL	A0A0B4SX31	*Cannabis sativa*	312	15	4.1.1.39	carboxylation of D-ribulose 1,5-bisphosphate	Photosynthesis
Small ubiquitin-related modifier	smt3	Q5TIQ0	*Cannabis sativa*	76	2		protein sumoylation	Protein metabolism
Cytochrome c biogenesis FC	ccmFc	A0A0M4RVN1	*Cannabis sativa*	447	1		Mitochondrial electron carrier protein	Respiration
Cytochrome c biogenesis FN	ccmFn	A0A0M3UM18	*Cannabis sativa*	575	2		Mitochondrial electron carrier protein	Respiration
Cytochrome c biogenesis protein CcsA	ccsA	A0A0C5B2L0	*Cannabis sativa*	320	1		biogenesis of c-type cytochromes	Respiration
Cytochrome c	cytC	P00053	*Cannabis sativa*	111	2		Mitochondrial electron carrier protein	Respiration
7S vicilin-like protein	Cs7S	A0A219D1T7	*Cannabis sativa*	493	2		nutrient reservoir activity	Storage
Edestin 1	ede1D	A0A090CXP5	*Cannabis sativa*	511	1		Seed storage protein	Storage
4-(cytidine 5’-diphospho)-2-C-methyl-D-erythritol kinase	CMK	A0A1V0QSI2	*Cannabis sativa*	408	4		Adds 2-phosphate to 4-CDP-2-C-methyl-d-erythritol	Terpenoid biosynthesis
1-deoxy-D-xylulose-5-phosphate synthase	DXPS1	A0A1V0QSH6	*Cannabis sativa*	730	2		Converts d-glyceraldehyde 3P into 1-deoxy-d-xylulose 5P	Terpenoid biosynthesis
1-deoxy-D-xylulose-5-phosphate synthase	DXS2	A0A1V0QSH5	*Cannabis sativa*	606	5		Converts d-glyceraldehyde 3P into 1-deoxy-d-xylulose 5P	Terpenoid biosynthesis
4-hydroxy-3-methylbut-2-en-1-yl diphosphate synthase	HDS	A0A1V0QSG3	*Cannabis sativa*	748	3		Converts (E)-4-hydroxy-3-methylbut-2-en-1-yl-2P into 2-C-methyl-d-erythritol 2,4-cyclo-2P	Terpenoid biosynthesis
3-hydroxy-3-methylglutaryl coenzyme A reductase	hmgR	A0A1V0QSF5	*Cannabis sativa*	588	5	1.1.1.34	synthesizes (R)-mevalonate from acetyl-CoA	Terpenoid biosynthesis
3-hydroxy-3-methylglutaryl coenzyme A reductase	hmgR	A0A1V0QSG7	*Cannabis sativa*	572	2	1.1.1.34	synthesizes (R)-mevalonate from acetyl-CoA	Terpenoid biosynthesis
Terpene synthase	TPS	A0A1V0QSF2	*Cannabis sativa*	567	1		formation of cyclic terpenes through the cyclisation of linear terpenes	Terpenoid biosynthesis
Terpene synthase	TPS	A0A1V0QSF3	*Cannabis sativa*	551	3		formation of cyclic terpenes through the cyclisation of linear terpenes	Terpenoid biosynthesis
Terpene synthase	TPS	A0A1V0QSF4	*Cannabis sativa*	613	1		formation of cyclic terpenes through the cyclisation of linear terpenes	Terpenoid biosynthesis
Terpene synthase	TPS	A0A1V0QSF6	*Cannabis sativa*	551	1		formation of cyclic terpenes through the cyclisation of linear terpenes	Terpenoid biosynthesis
Terpene synthase	TPS	A0A1V0QSF8	*Cannabis sativa*	629	2		formation of cyclic terpenes through the cyclisation of linear terpenes	Terpenoid biosynthesis
Terpene synthase	TPS	A0A1V0QSF9	*Cannabis sativa*	624	2		formation of cyclic terpenes through the cyclisation of linear terpenes	Terpenoid biosynthesis
Terpene synthase	TPS	A0A1V0QSG0	*Cannabis sativa*	573	1		formation of cyclic terpenes through the cyclisation of linear terpenes	Terpenoid biosynthesis
Terpene synthase	TPS	A0A1V0QSG1	*Cannabis sativa*	640	1		formation of cyclic terpenes through the cyclisation of linear terpenes	Terpenoid biosynthesis
Terpene synthase	TPS	A0A1V0QSG6	*Cannabis sativa*	556	3		formation of cyclic terpenes through the cyclisation of linear terpenes	Terpenoid biosynthesis
Terpene synthase	TPS	A0A1V0QSH1	*Cannabis sativa*	594	1		formation of cyclic terpenes through the cyclisation of linear terpenes	Terpenoid biosynthesis
(-)-limonene synthase, chloroplastic	TPS1	A7IZZ1	*Cannabis sativa*	622	2	4.2.3.16	monoterpene (C10) olefins biosynthesis	Terpenoid biosynthesis
Maturase K	matK	A0A1V0IS32	*Cannabis sativa*	509	1		assists in splicing its own and other chloroplast group II intron	Transcription
Maturase K	matK	Q95BY0	*Cannabis sativa*	507	2		assists in splicing its own and other chloroplast group II intron	Transcription
Maturase R	matR	A0A0M5M254	*Cannabis sativa*	651	1		assists in splicing introns	Transcription
DNA-directed RNA polymerase subunit beta	rpoB	A0A0C5ARQ8	*Cannabis sativa*	1070	3	2.7.7.6	transcription of DNA into RNA	Transcription
DNA-directed RNA polymerase subunit beta	rpoB	A0A0C5ARX9	*Cannabis sativa*	1393	4	2.7.7.6	transcription of DNA into RNA	Transcription
DNA-directed RNA polymerase subunit beta	rpoB	A0A0U2H5U7	*Cannabis sativa*	1070	1	2.7.7.6	transcription of DNA into RNA	Transcription
DNA-directed RNA polymerase subunit beta	rpoC1	A0A0C5AUF5	*Cannabis sativa*	683	6	2.7.7.6	transcription of DNA into RNA	Transcription
DNA-directed RNA polymerase subunit beta	rpoC2	A0A0H3W6G1	*Cannabis sativa*	1389	1	2.7.7.6	transcription of DNA into RNA	Transcription
DNA-directed RNA polymerase subunit beta	rpoC2	A0A0X8GKF1	*Cannabis sativa*	1391	1	2.7.7.6	transcription of DNA into RNA	Transcription
DNA-directed RNA polymerase subunit beta	rpoC2	A0A1V0IS28	*Cannabis sativa*	1393	1	2.7.7.7	transcription of DNA into RNA	Transcription
Ribosomal protein L14	rpl14	A0A0C5AS10	*Cannabis sativa*	122	2		assembly of the ribosome	Protein metabolism
50S ribosomal protein L16, chloroplastic	rpl16	A0A0C5AUJ2	*Cannabis sativa*	119	2		assembly of the 50S ribosomal subunit	Protein metabolism
Ribosomal protein L2	rpl2	A0A0M3ULW5	*Cannabis sativa*	337	2		assembly of the ribosome	Protein metabolism
50S ribosomal protein L20	rpl20	A0A0C5B2J3	*Cannabis sativa*	120	1		Binds directly to 23S rRNA to assemble the 50S ribosomal subunit	Protein metabolism
Ribosomal protein S11	rps11	A0A0C5ART4	*Cannabis sativa*	138	1		assembly of the ribosome	Protein metabolism
30S ribosomal protein S12, chloroplastic	rps12	A0A0C5APY5	*Cannabis sativa*	132	1		translational accuracy	Protein metabolism
30S ribosomal protein S12, chloroplastic	rps12	A0A0C5B2L8	*Cannabis sativa*	125	1		translational accuracy	Protein metabolism
Ribosomal protein S13	rps13	A0A0M5M201	*Cannabis sativa*	116	1		assembly of the ribosome	Protein metabolism
Ribosomal protein S19	rps19	A0A0M3ULW7	*Cannabis sativa*	94	1		assembly of the ribosome	Protein metabolism
Ribosomal protein S2	rps2	A0A0C5APX8	*Cannabis sativa*	236	1		assembly of the ribosome	Protein metabolism
30S ribosomal protein S3, chloroplastic	rps3	A0A0C5ART6	*Cannabis sativa*	155	3		assembly of the 30S ribosomal subunit	Protein metabolism
Ribosomal protein S3	rps3	A0A0M3UM22	*Cannabis sativa*	548	1		assembly of the ribosome	Protein metabolism
Ribosomal protein S3	rps3	A0A110BC84	*Cannabis sativa*	548	1		assembly of the ribosome	Protein metabolism
Ribosomal protein S4	rps4	A0A0M4RG21	*Cannabis sativa*	352	1		assembly of the ribosome	Protein metabolism
Ribosomal protein S7	rps7	A0A0C5ARU3	*Cannabis sativa*	155	2		assembly of the ribosome	Protein metabolism
Ribosomal protein S7	rps7	A0A0M4R6T5	*Cannabis sativa*	148	1		assembly of the ribosome	Protein metabolism
Protein TIC 214	ycf1	A0A0C5AS14	*Cannabis sativa*	356	2		protein precursor import into chloroplasts	Protein metabolism
Protein TIC 214	ycf1	A0A0H3W815	*Cannabis sativa*	1878	21		protein precursor import into chloroplasts	Protein metabolism
Acyl-activating enzyme 1	aae1	H9A1V3	*Cannabis sativa*	720	1			Unknown
Acyl-activating enzyme 10	aae10	H9A1W2	*Cannabis sativa*	564	1			Unknown
Acyl-activating enzyme 12	aae12	H9A8L1	*Cannabis sativa*	757	2			Unknown
Acyl-activating enzyme 13	aae13	H9A8L2	*Cannabis sativa*	715	3			Unknown
Acyl-activating enzyme 2	aae2	H9A1V4	*Cannabis sativa*	662	3			Unknown
Acyl-activating enzyme 3	aae3	H9A1V5	*Cannabis sativa*	543	7			Unknown
Acyl-activating enzyme 4	aae4	H9A1V6	*Cannabis sativa*	723	3			Unknown
Acyl-activating enzyme 5	aae5	H9A1V7	*Cannabis sativa*	575	1			Unknown
Acyl-activating enzyme 6	aae6	H9A1V8	*Cannabis sativa*	569	1			Unknown
Acyl-activating enzyme 8	aae8	H9A1W0	*Cannabis sativa*	526	3			Unknown
Cannabidiolic acid synthase-like 2	CBDAS3	A6P6W1	*Cannabis sativa*	545	1		Has no cannabidiolic acid synthase activity	Unknown
Putative LOV domain-containing protein	LOV	A0A126WVX7	*Cannabis sativa*	664	8			Unknown
Putative LOV domain-containing protein	LOV	A0A126WVX8	*Cannabis sativa*	1063	7			Unknown
Putative LOV domain-containing protein	LOV	A0A126WZD3	*Cannabis sativa*	574	1			Unknown
Putative LOV domain-containing protein	LOV	A0A126X0M1	*Cannabis sativa*	725	4			Unknown
Putative LOV domain-containing protein	LOV	A0A126X1H2	*Cannabis sativa*	910	6			Unknown
Putative LysM domain containing receptor kinase	lyk2	U6EFF4	*Cannabis sativa*	599	1			Unknown
Uncharacterised protein	unknown	A0A1V0IS79	*Cannabis sativa*	1525	2			Unknown
Uncharacterised protein	unknown	L0N5C8	*Cannabis sativa*	543	1			Unknown
Protein Ycf2	ycf2	A0A0C5APZ4	*Cannabis sativa*	2302	9		ATPase of unknown function	Unknown
Protein translocase subunit	secA	A0A0N9ZJA6	*Cannabis sativa*	158	7		Binds ATP	Protein metabolism
ATP synthase subunit beta, chloroplastic	atpB	A0A0U2DTF2	*Cannabis sativa subsp. sativa*	498	20	3.6.3.14	Produces ATP from ADP	Energy metabolism
Acetyl-coenzyme A carboxylase carboxyl transferase subunit beta, chloroplastic	accD	A0A0U2DTG7	*Cannabis sativa subsp. sativa*	497	3	2.1.3.15	acetyl coenzyme A carboxylase complex	Lipid biosynthesis
NAD(P)H-quinone oxidoreductase subunit K, chloroplastic	ndhK	A0A0U2DTF9	*Cannabis sativa subsp. sativa*	226	1	1.6.5.-	NDH shuttles electrons from NAD(P)H:plastoquinone to quinones	Photosynthesis
Cytochrome f	petA	A0A0U2DW83	*Cannabis sativa subsp. sativa*	320	1		mediates electron transfer between PSII and PSI	Photosynthesis
Photosystem II protein D1	psbA	A0A0U2DTE4	*Cannabis sativa subsp. sativa*	353	2	1.10.3.9	assembly of the PSII complex	Photosynthesis
Photosystem II CP43 reaction center protein	psbC	A0A0U2DTE2	*Cannabis sativa subsp. sativa*	473	5		core complex of PSII	Photosynthesis
Photosystem II D2 protein	psbD	A0A0U2DVP6	*Cannabis sativa subsp. sativa*	353	3	1.10.3.9	assembly of the PSII complex	Photosynthesis
Cytochrome b559 subunit alpha	psbE	A0A0U2DTH9	*Cannabis sativa subsp. sativa*	83	2		reaction center of PSII	Photosynthesis
Ribulose bisphosphate carboxylase large chain	rbcL	A0A0U2DW50	*Cannabis sativa subsp. sativa*	475	13	4.1.1.39	carboxylation of D-ribulose 1,5-bisphosphate	Photosynthesis
Photosystem I assembly protein Ycf4	ycf4	A0A0U2DVM4	*Cannabis sativa subsp. sativa*	184	1		assembly of the PSI complex	Photosynthesis
30S ribosomal protein S14, chloroplastic	rps14	A0A0U2DTI4	*Cannabis sativa subsp. sativa*	100	2		Binds 16S rRNA, required for the assembly of 30S particles	Protein metabolism
30S ribosomal protein S15, chloroplastic	rps15	A0A0U2DW79	*Cannabis sativa subsp. sativa*	90	1		assembly of the 30S ribosomal subunit	Protein metabolism
ATP synthase subunit beta, chloroplastic	atpB	A0A0U2H0U7	*Humulus lupulus*	498	2	3.6.3.14	Produces ATP from ADP	Energy metabolism
ATP synthase subunit beta, chloroplastic	atpB	A0A0U2H587	*Humulus lupulus*	191	1		Component of the F(0) channel	Energy metabolism
NAD(P)H-quinone oxidoreductase subunit I, chloroplastic	ndhI	A0A0U2GY49	*Humulus lupulus*	171	2	1.6.5.-	NDH shuttles electrons from NAD(P)H:plastoquinone to quinones	Photosynthesis
DNA-directed RNA polymerase subunit beta	rpoC2	A0A0U2H146	*Humulus lupulus*	1398	1	2.7.7.6	transcription of DNA into RNA	Transcription
50S ribosomal protein L20, chloroplastic	rpl20	A0A0U2H0V8	*Humulus lupulus*	120	1		Binds directly to 23S rRNA to assemble the 50S ribosomal subunit	Protein metabolism
30S ribosomal protein S4, chloroplastic	rps4	A0A0U2H5A0	*Humulus lupulus*	202	1		binds directly to 16S rRNA to assemble the 30S subunit	Protein metabolism
30S ribosomal protein S8, chloroplastic	rps8	A0A0U2GZU5	*Humulus lupulus*	134	2		binds directly to 16S rRNA to assemble the 30S subunit	Protein metabolism
Protein Ycf2	ycf2	A0A0U2H6B6	*Humulus lupulus*	2287	1		ATPase of unknown function	Unknown

## Supplementary Materials

The following are available online, Figure S1: LC-MS maps of intact proteins, Figure S2: NanoLC-MS maps of tryptic peptides, Figure S3: Distribution of *Cannabis sativa* entries in UniprotKB from 1986 to 2018, Figure S4: Quantitative comparison of the six extraction methods. Table S1: Bottom-up identification results for Bovine Serum Albumin (BSA) sample (two injection replicates), Table S2: Number of nLC-MS peaks detected and clusters quantified in Genedata Expressionist Refiner across the various extraction methods. Table S3: List of peptides and proteins identified in cannabis apical buds and trichomes by bottom-up proteomics.
