# Supplementary material for: Optimisation of Protein Extraction from Medicinal Cannabis Mature Buds for Bottom-Up Proteomics
Source: Molecules. 2019 Feb 13;24(4):659. doi: 10.3390/molecules24040659 (PMC6412734; doi:10.3390/molecules24040659)

Supplementary Figure S1: LC-MS maps of intact proteins.

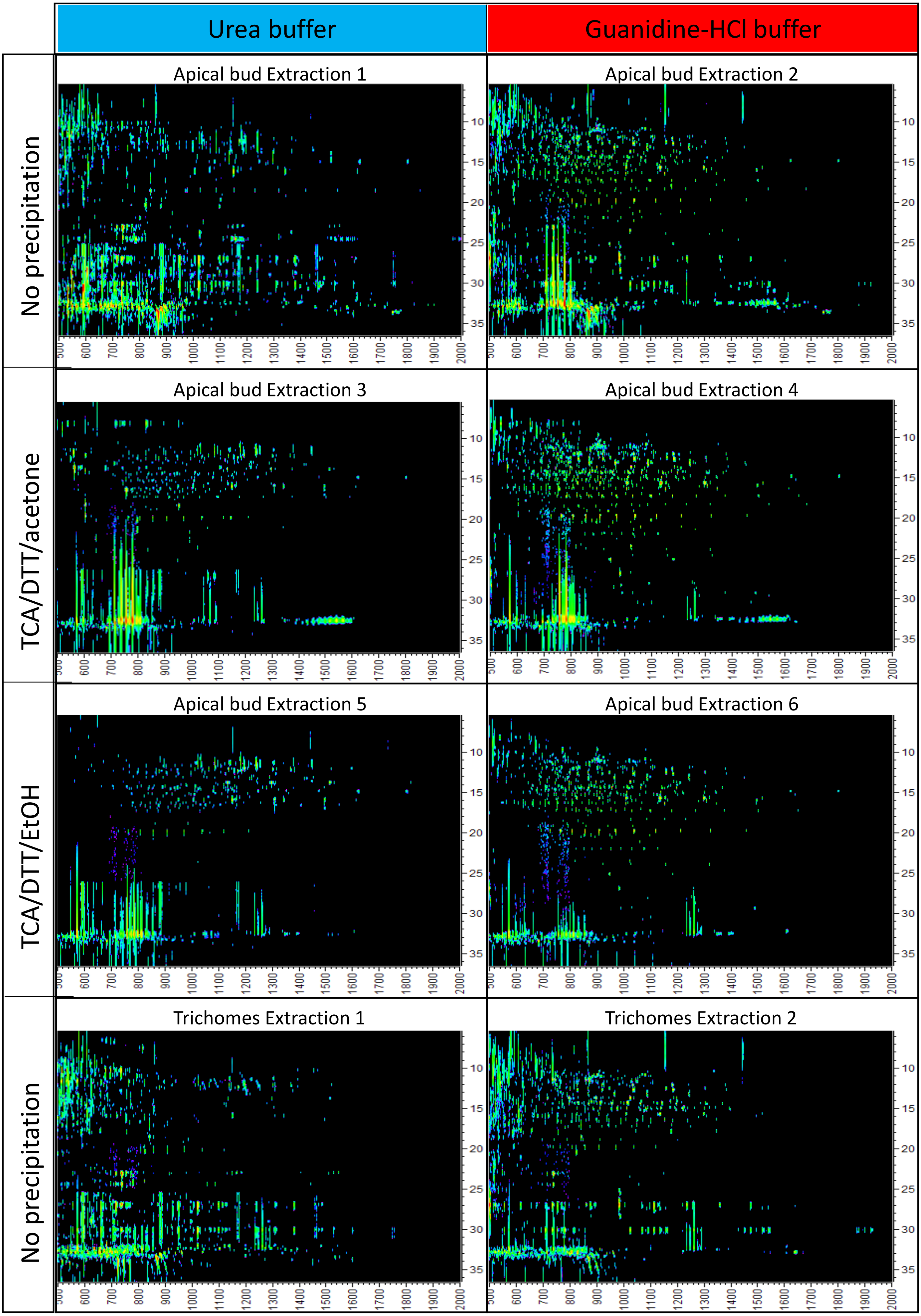

Supplementary Figure S2: **nLC-MS maps of tryptic peptides.**

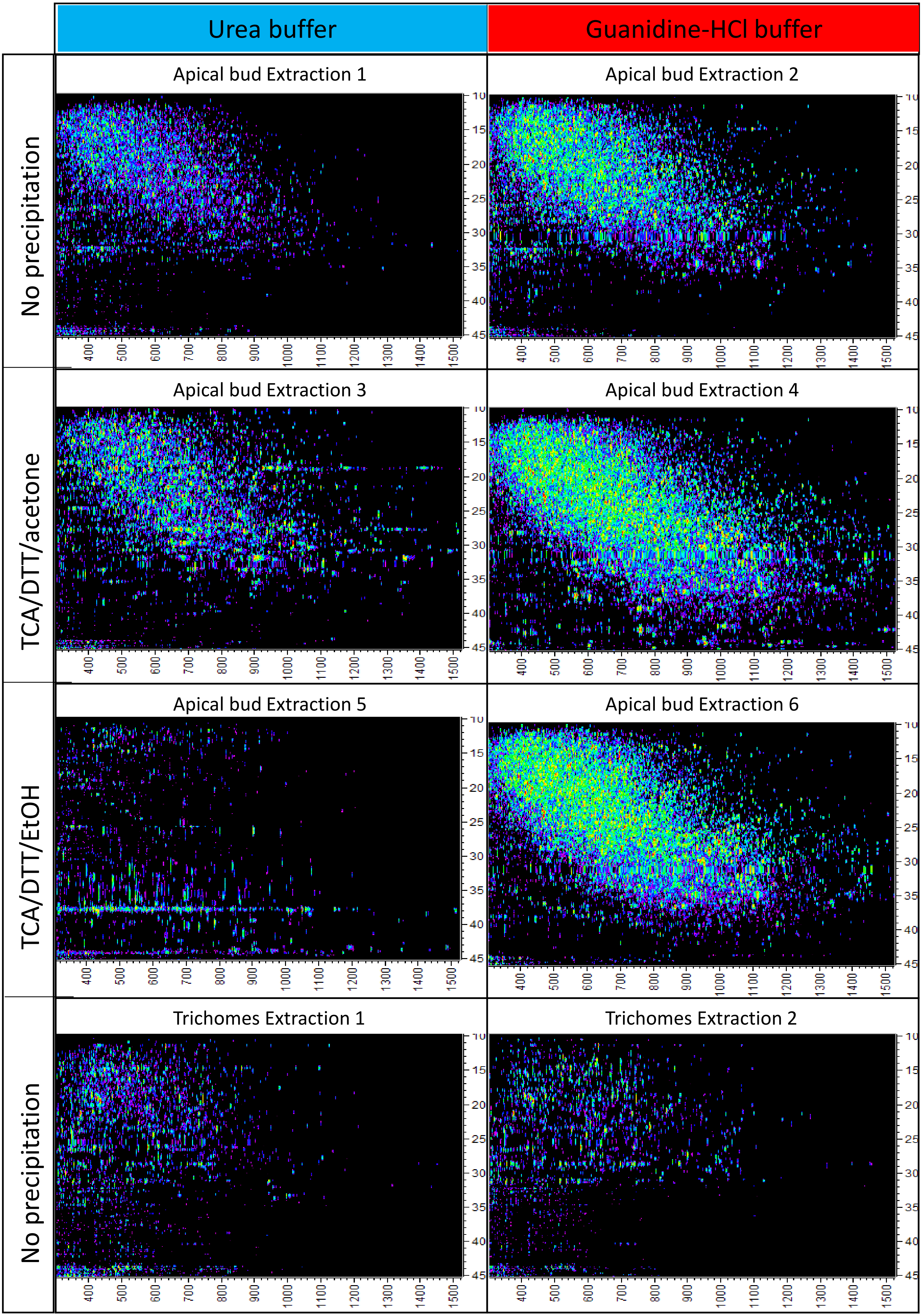

<u>Supplementary Figure S3:</u> Distribution of Cannabis sativa entries in UniprotKB from 1986 to 2018.

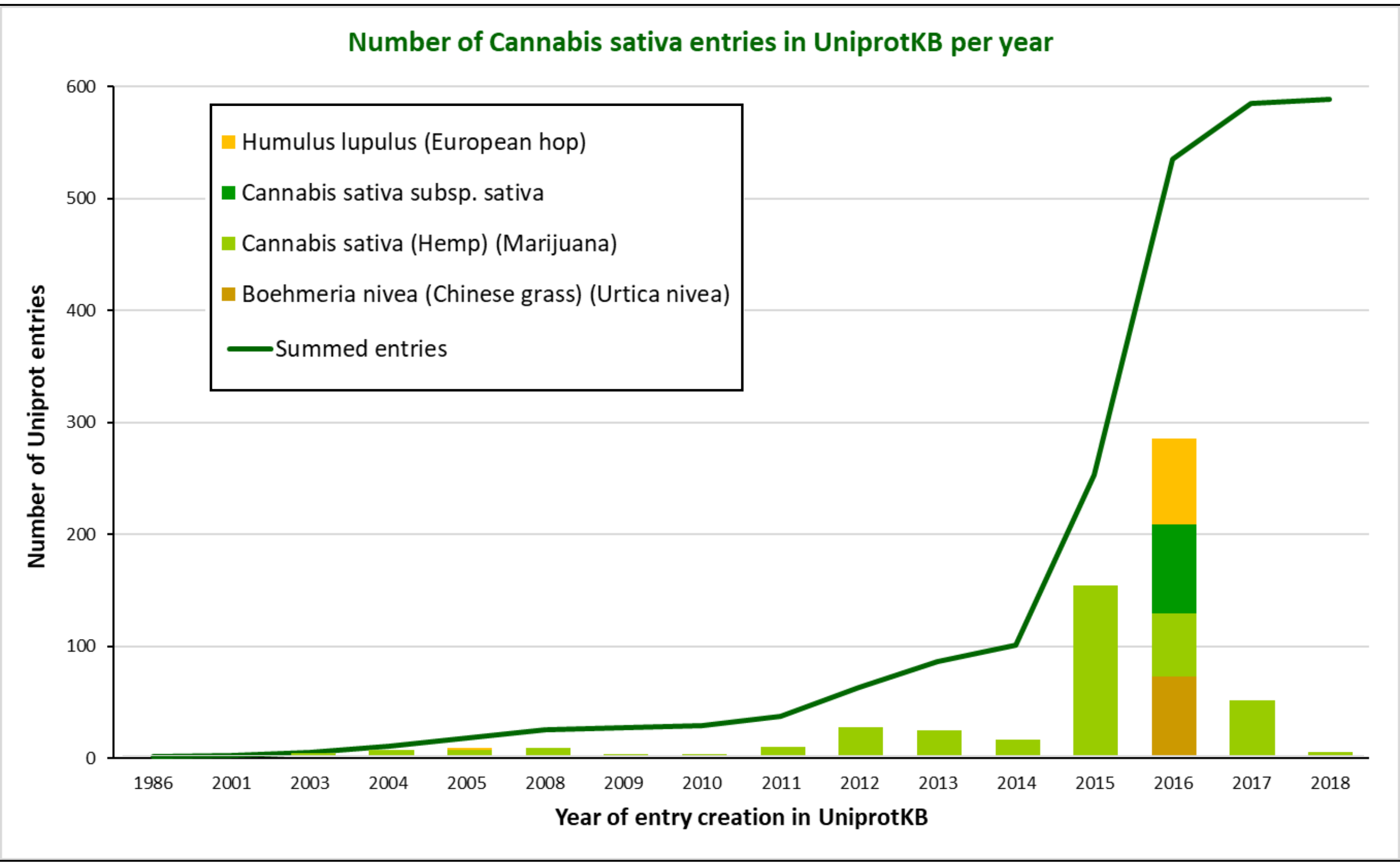

<u>Supplementary Figure S4</u>: Quantitative comparison of the six extraction methods.
Values obtained number of intact proteins quantified by UPLC-MS and number of tryptic peptides identified by nLC-MS/MS were converted into percent and coefficient of variation (CV). Resulting percentages are plotted in the histogram below and vertical bars denote CVs.

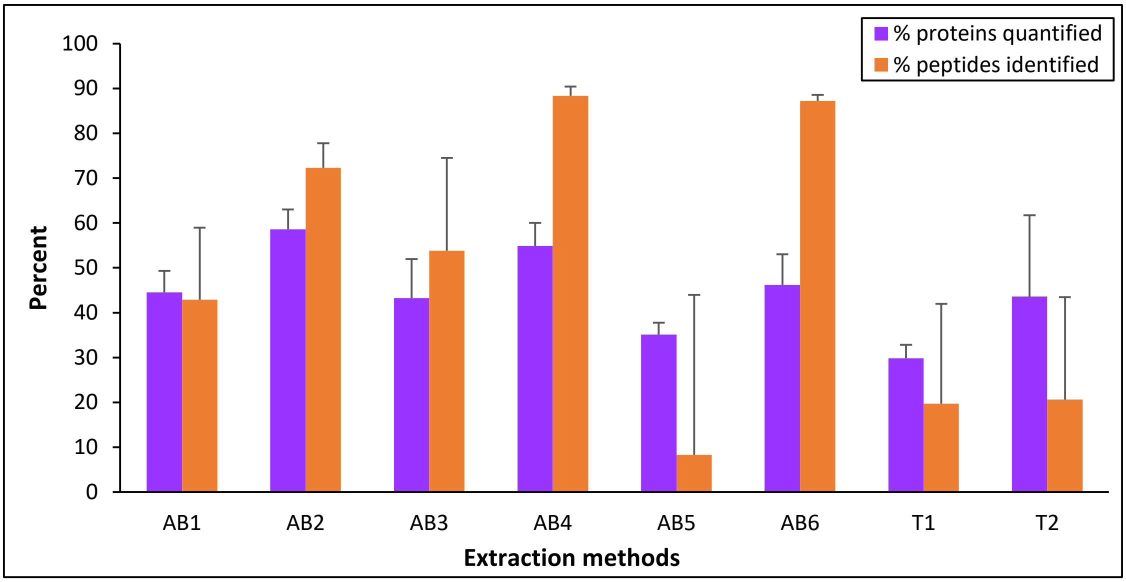

Supplement: Supplementary file 1 [file molecules-24-00659-s001.zip › MC_protein optimisation paper_SupplFig-S1-S4.pdf]
